# Chaperones and Catalysts: How Antigen Presentation Pathways Cope With Biological Necessity

**DOI:** 10.3389/fimmu.2022.859782

**Published:** 2022-04-07

**Authors:** David H. Margulies, Daniel K. Taylor, Jiansheng Jiang, Lisa F. Boyd, Javeed Ahmad, Michael G. Mage, Kannan Natarajan

**Affiliations:** Laboratory of Immune System Biology, National Institute of Allergy and Infectious Diseases, Molecular Biology Section, National Institutes of Health, Bethesda, MD, United States

**Keywords:** Antigen presentation, peptide loading complex (PLC), tapasin, TAP binding protein, related (TAPBPR), major histocompatibility complex (MHC), protein folding, structural immunology

## Abstract

Immune recognition by T lymphocytes and natural killer (NK) cells is in large part dependent on the identification of cell surface MHC molecules bearing peptides generated from either endogenous (MHC I) or exogenous (MHC II) dependent pathways. This review focuses on MHC I molecules that coordinately fold to bind self or foreign peptides for such surface display. Peptide loading occurs in an antigen presentation pathway that includes either the multimolecular peptide loading complex (PLC) or a single chain chaperone/catalyst, TAP binding protein, related, TAPBPR, that mimics a key component of the PLC, tapasin. Recent structural and dynamic studies of TAPBPR reveal details of its function and reflect on mechanisms common to tapasin. Regions of structural conservation among species suggest that TAPBPR and tapasin have evolved to satisfy functional complexities demanded by the enormous polymorphism of MHC I molecules. Recent studies suggest that these two chaperone/catalysts exploit structural flexibility and dynamics to stabilize MHC molecules and facilitate peptide loading.

## Introduction

Classical experiments indicate that proteins arrive at their stable three-dimensional conformation at their lowest Gibbs free energy, achieved as a result of their primary amino acid sequence and their interactions with solvent (1, 2). Nevertheless, the potential timescale of searching the myriad possible conformations of a protein as noted by Levinthal (3, 4) raised a conundrum solved only partially by the recognition of the contribution of protein nucleation regions and folding landscapes (5, 6) to the descent along an energy funnel to achieve a final stable structure (7). More recently, the so-called “protein folding problem” has been redefined in terms of the practical utility of predicting a protein’s three-dimensional structure from its primary amino acid sequence. This computational boundary is now being overcome by the concurrence of large and ever increasing structural and sequence databases with innovative artificial intelligence approaches by DeepMind and its implementation of AlphaFold2 (8). However, by contrast to the apparent success of structure prediction of individual proteins in recent years, our understanding of the rules that govern protein interactions remain rudimentary. To paraphrase Donne (9), no protein is an island. During the course of its lifetime, from biogenesis on the ribosome to destruction by the proteasome, a single protein molecule must interact with a multitude of partners. These include chaperones that aid its folding and prevent aggregation, enzymes that add post-translational modifications, transport proteins that escort it to its destinations, the substrate on which it performs its biological function, and the ubiquitinylating enzymes that target it for destruction. The evolutionarily conserved, and crucially important, antigen presentation pathway in vertebrates provides a valuable model system in which to investigate these various events in the life of a protein

Reflected in the pathways that have evolved to permit coassembly of antigenic peptides with their glycoprotein antigen presenting elements, the antigen presentation pathways that govern the biosynthesis, folding, assembly, peptide loading, peptide exchange, and cell surface expression of peptide/protein complexes are crucial to the immune response to tumors, viruses, and a variety of cellular pathogens (10–12). These pathways are based on the major histocompatibility complex (MHC) encoded class I (MHC I) and class II (MHC II) proteins, and their associated molecules. In this speculative review, we will focus on the classical MHC I molecules, HLA-A, -B, and -C in the human and H2-K, -D, and -L in the mouse, obligate cell surface intrinsic membrane proteins, that serve as recognition elements for T cell receptors (TCR) expressed on CD8+ T lymphocytes as well as ligands for various receptors on natural killer (NK) cells and other hematopoietic effector cells.

## MHC Molecules, Not All Are the Same

The most remarkable characteristic of classical MHC I molecules is that they are highly polymorphic. That is, the number of allelomorphic variants in the human population, encoded at the three major genetic loci, *HLA-A, -B, and -C*, is enormous, catalogued by the IMGT database to be greater than 22,000 at current count (13). These are cell surface expressed type I membrane glycoproteins that are complexed with an essentially monomorphic light chain, β_2_-microglobulin (β_2_m). In addition, each MHC I molecule of a given cell binds a multitude of peptides derived from an endogenous MHC I pathway, thus generating a large repertoire of surface molecules available for interaction with immune cell receptors. The puzzles of course, are how do all these distinct MHC I molecules fold, how does each one form a stable ternary complex bound to each of thousands of potential peptides, and how does the biological system select for the most thermodynamically stable peptide/MHC I complexes for display at the cell surface.

## The Peptide Loading Complex – A Molecular Machine for MHC I Assembly and Peptide Loading

**Figure 1:**
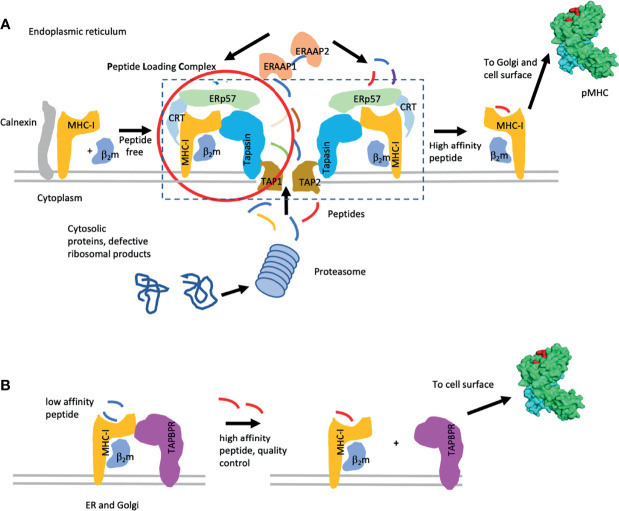
Pathways of MHC-I peptide loading. **(A)** Classic MHC-I peptide loading pathway is illustrated. Components of the pathway [i.e. the peptide loading complex (PLC)] include the chaperones calnexin, calreticulin (CRT), ERp57, tapasin, and the TAP1/2 peptide transporter. Peptides are indicated. Additional quality control through the UGT pathway is described in the text. **(B)** Peptide exchange with the chaperone TAPBPR are indicated. Lower affinity peptides are replaced by higher affinity peptides while the MHC I molecules are stabilized by either tapasin or TAPBPR. When high affinity peptide is bound, the MHC I complex dissociates from the chaperone and proceeds to the cell surface. [This figure is a modification of one published elsewhere (16)].

Several decades of experimentation have identified the peptide loading complex (PLC), a multimolecular dynamic machine that sequentially stabilizes the MHC I heavy chain to fold with its light chain β_2_m, then to access and bind antigenic peptides delivered to the lumen of the endoplasmic reticulum, to exchange and evaluate peptides to identify the best binders, to pass quality control, to access the *cis* Golgi, and to proceed from there to the cell surface (10–12). Major insights included the identification of the roles of the chaperone/lectins calnexin and calreticulin that monitor the sequential glycosylation of the MHC I heavy chain. Further studies recognized the importance of the transporter associated with antigen processing (TAP) 1 and 2, an ATP-dependent heterodimer that delivers peptides from the proteasome-generated cytoplasmic pool to the ER, and the crucial function of tapasin, an ER protein that bridges TAP to the nascently folding/peptide binding MHC I/β_2_m complex, and an oxidoreductase, ERp57. Additional steps in the quality control of peptide-loaded MHC I include glycan-dependent interactions (14, 15). These steps of the classical peptide loading pathway are illustrated in **Figure 1**.

Visualization of structural aspects of tapasin function was first achieved in a classical paper by Dong, Wearsch and colleagues (17), which reported the X-ray crystallographic structure of human tapasin bound to ERp57. This work, complemented by mutational analysis of MHC I molecules and study of MHC I polymorphic variants, provided several molecular models for how tapasin interacts with MHC I, revealing how it might stabilize partially folded MHC I and encourage peptide exchange (18–22).

**Figure 2:**
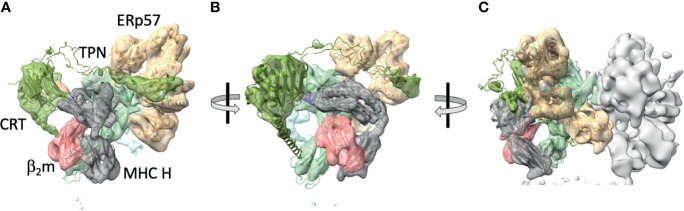
Cryo-EM maps of the complete protein loading complex reveal spatial organization of its components. **(A)** 5.8 Å cryo-EM map of the PLC editing module (EMD-3906) with superposed X-ray -derived models (PDB-6ENY) as described by (23). **(B)** PLC rotated approximately 30° to center the β_2_m/MHC H chain module. **(C)** 7.2 Å cryo-EM map of the pseudosymmetrical PLC module, with the left side editing module colored and the right side one illustrating by map surface alone. Map and models with generated with ChimeraX. MHC H chain is grey, β_2_m is pink, calreticulin is green, tapasin is pale blue, and ERp57 is tan.

In the absence of detailed structural information on the nature of the tapasin/MHC I association, a cryo-electron microscopic approach was taken by Blees et al, who established a three-dimensional view, albeit at modest resolution (7.2 Å for the full complex, 5.8 Å for the editing module). This established the relationships between the components of the PLC: β_2_m, MHC I heavy chain, TAP, tapasin, ERp57, and calreticulin, and confirmed the stoichiometry previously established by pull-down experiments (23, 24) (**Figure 2**). Thus, the full PLC was visualized as containing one TAP1/2 heterodimer, with each chain flanked by an MHC I/β2m/ERp57/calreticulin complex (see **Figure 2**). Visualization of peptide was difficult at this resolution.

## TAPBPR a Surrogate PLC, Reveals Further Details of CHAPERONE/CATALYST Function

As the complete map of the human genome became available, several groups identified genetic regions paralagous to the extended major histocompatibility complex (25, 26) and Teng et al. identified a gene encoding a tapasin-like molecule (27). Studies of the encoded protein languished until Boyle et al. (28) demonstrated an interaction between TAPBPR and MHC I, independent of other components of the PLC. Further studies not only confirmed the potential for MHC I association, but also established both chaperone and catalytic activities of TAPBPR that mimicked tapasin. Although the precise biological necessity for TAPBPR remains unclear (29), some novel functions, including control of trafficking to the UDP-glucose:glycoprotein glucosyltransferase quality control pathway have been observed (30). Additionally, TAPBPR interactions with MHC I are quantitatively dependent on the glycosylation status of the MHC I molecule (31, 32). In addition, TAPBPR distinguishes different MHC molecules based on their polymorphism (33, 34). Recently, exploitation of the catalytic peptide exchange functions of TAPBPR have given rise to new technologies facilitating the production of recombinant MHC I molecules (35–37).

Structural studies of TAPBPR have offered insight not only into its own function, but also to that of the tapasin homolog (38–40). Initial low resolution small angle X-ray scattering analysis (34) comparing recombinant tapasin with TAPBPR revealed their structural similarity as predicted by their shared amino acid sequences (41, 42).

**Figure 3:**
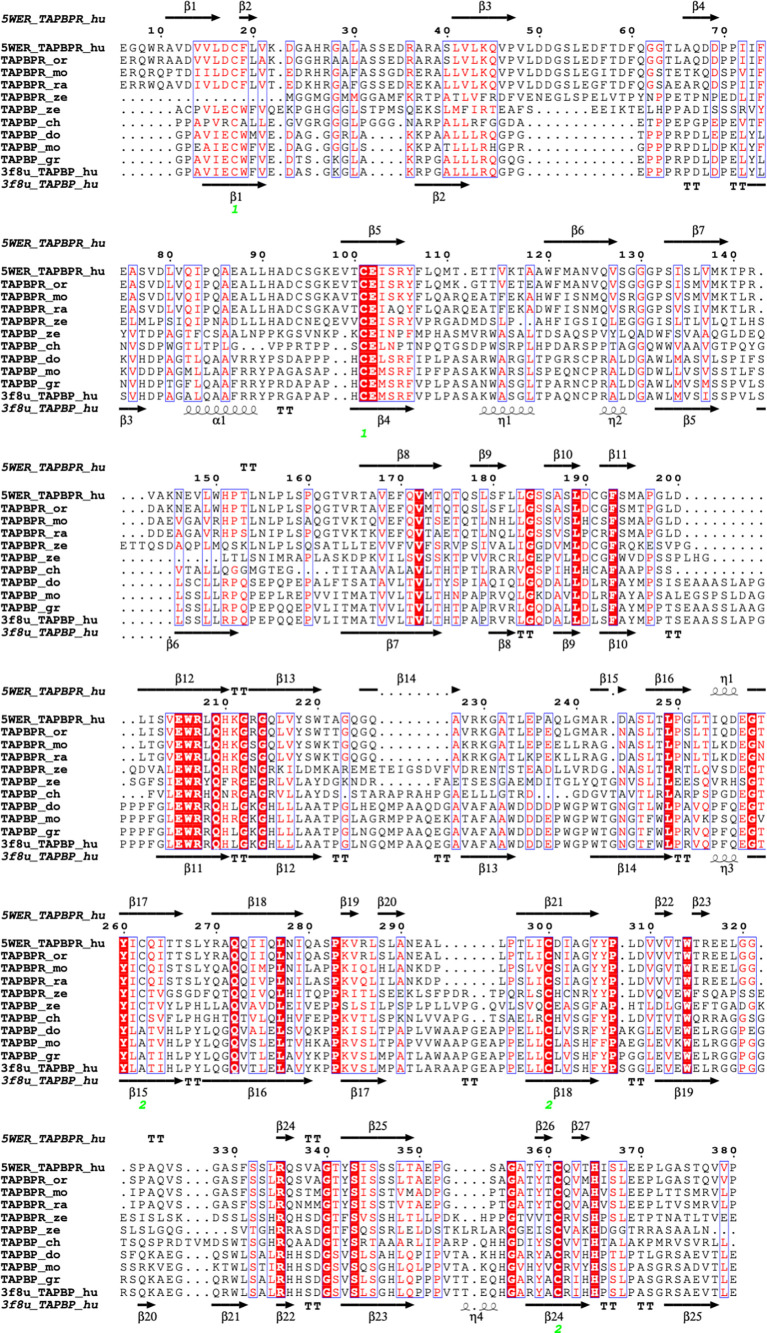
Structure based amino acid sequence alignment of TAPBPR and tapasin (TAPBP) sequences from several different species. Sequences were aligned with ClustalW in MacVector 18.0 and further illustrated with ESPript on the ENDscript server (57). Top and bottom sequences and secondary structure elements are taken from TAPBPR (5WER) and tapasin (TAPBP) (3F8U) respectively. Sequences of TAPBPR_or (orangutan-Pongo abellii-Q5R8H1), TAPBPR_mo (mouse-Mus musculus-Q8VD31), TAPBPR_ra (rat-Rattus novegicus-D4A6L1), and TAPBPR_ze (zebrafish-Danio rerio-X1WBD6), as well as TAPBP_gr (gorilla-Chlorocebus aethiops-Q6PZD2), TAPBP_do (dog-Canis lupus familiaris-Q5TJE4), TAPBP_ch (chicken-Gallus gallus-Q9R233), and TAPBP_ze (zebrafish-Danio rerio-Q1LUU3) are provided.

These results with TAPBPR suggested that higher resolution structure determination of TAPBPR might offer further insight into the mechanism by which TAPBPR, and by inference tapasin, function in their dual roles as chaperones and catalysts. Two reports of X-ray structures of MHC I/TAPBPR complexes were reported at the same time—one of a complex of the mouse MHC I molecule H2-D^b^ complexed with human TAPBPR (39), and another of the mouse H2-D^d^ with human TAPBPR (38). The models derived in both laboratories are remarkably similar (rmsd for the superposition of the TAPBPR/MHC I/β_2_m complexes was 1.158 Å for 3385 atoms). The H2-D^b^ complex was generated with H2-D^b^ emptied of a labile peptide by photolysis, and the H2-D^d^ complex was generated with a covalently-linked truncated peptide. Nevertheless, in both structures, no peptide was visualized. (For the covalent peptide/H2-D^d^ complex, it is presumed that this is due to structural heterogeneity or mobility of the peptide moiety.) The two structures were in remarkable agreement, with the exception that a peptide loop representing residues K22-D35 of TAPBPR was modeled for the H2-D^b^ complex, while in the absence of reliable electron density in this region, no model was built for the H2-D^d^/TAPBPR complex (38). Critical assessment of whether there is solid evidence for such a loop has been presented elsewhere (43, 44). It is also relevant to consider the alignment of a selection of TAPBPR sequences from several species as compared with those of tapasin (**Figure 3**). Notably, the K22 -D35 loop is significantly longer in all TAPBPR molecules as compared with tapasin (labelled here, TAPBP) molecules.

Indeed, several experimental lines of indirect evidence suggest a competitive role for this loop in protecting the peptide binding groove of the MHC during the process of binding and folding. These include mutational analyses of the loop in TAPBPR (47, 48) and structural studies of truncated peptides complexed with MHC molecules (49). However, more recent nuclear magnetic resonance studies suggest that the TAPBPR loop functions dynamically, forming a lid that modulates the access of peptides to the peptide binding groove (50–52). Other interactions of TAPBPR with the exterior aspects of the peptide binding domains of the MHC I α1 and α2 domains as well as the interaction of the membrane proximal IgC-like domain of TAPBPR with the membrane proximal α3/β_2_m unit of the MHC I molecule are evidence of a global disruption of the peptide binding groove (16, 38, 43).

**Figure 4:**
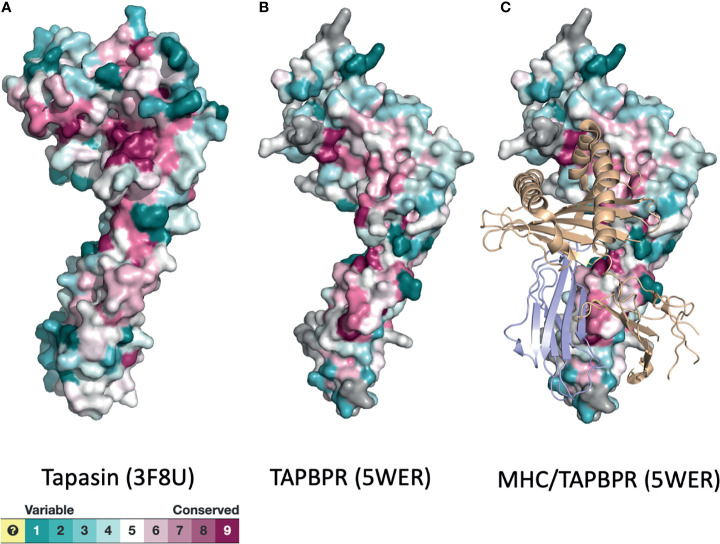
Surface representations of tapasin **(A)**, TAPBPR **(B)**, and MHC/TAPBPR complex **(C)** reveal regions of conservation and variability. PDB models of tapasin (3F8U) and TAPBPR (5WER) were submitted to the Consurf server (45, 46) to assess the evolutionary conservation of the amino acids of the indicated molecules. Color scheme for the degree of variability is shown.

These experimental findings on TAPBPR are complemented by molecular dynamics simulations of tapasin (44), MHC I molecules (53–55), and of a model of the entire PLC (56). These studies indicate that the chaperone/catalysts exhibit considerable flexibility to accommodate the structural plasticity of a wide range of peptide/MHC I complexes. As function is embodied in structure, amino acid sequence relationships over evolutionary time may be expected to reveal regions of tapasin or nTAPBPR that are conserved because of conserved function. In **Figure 4** we display the surface of X-ray structures of tapasin (**Figure 4A**) and TAPBPR (**Figure 4B**) colored according to their evolutionary variability as calculated with Consurf (45, 46). Although considerable variability may be noted, high degrees of conservation are observed in the region of the amino terminal domain of tapasin/TAPBPR that contacts MHC I (**Figure 4C**), as well as in the membrane proximal IgC domain of these molecules.

## Structure Predictions Across Species

**Figure 5:**
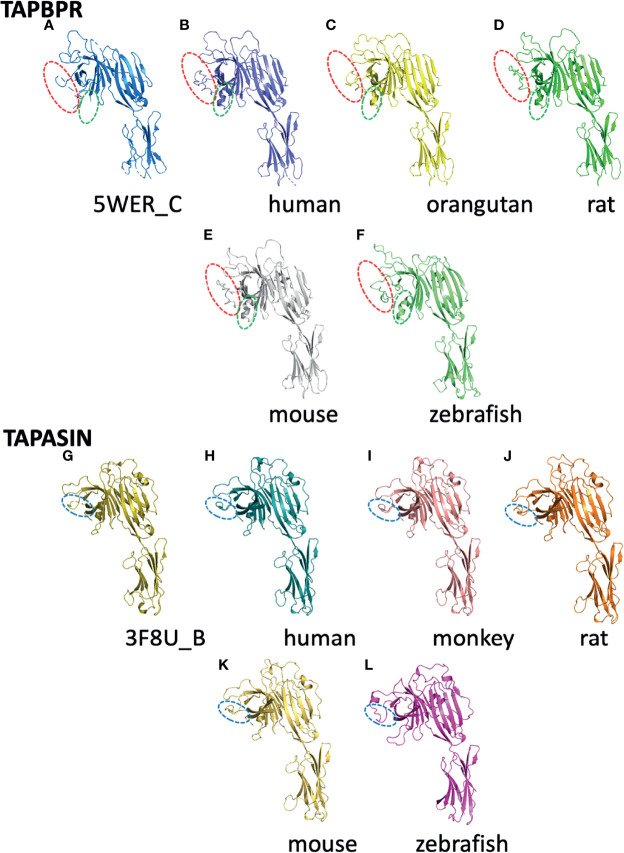
Structures predicted by AlphaFold2 preserve features of experimentally derived structures. The AlphaFold2 Protein Structure Database (https://alphafold.ebi.ac.uk) was queried for TAPBPL (alternate designation for TAPBPR) or TAPBP (for tapasin) and the resulting structural models were superposed on experimentally determined structures for TAPBPR (5WER chain C) **(A)** and tapasin (3F8U chain B) **(G)** as indicated. **(B)** represents the predicted human TAPBPR. **(C)** Pongo abelii (Sumatran orangutan). **(D)** Rattus norvegicus. **(E)** Mus musculus. **(F)** Danio rerio. Panels **(G–L)** represent tapasin (TAPBP) structure (3F8U chain B) **(G)**, and models of human **(H)**, Chlorocebus aethiops **(I)**, Rattus novegicus **(J)**, Mus musculus **(K)**, and Danio rerio **(L)**. As described in the text, loops that were not modeled based on X-ray data but were modeled by AlphaFold2 are indicated by dashed ovals.

The recent success of the application of AlphaFold2 to protein structure prediction (8, 58, 59) compels us to exploit this powerful approach to explore likely three-dimensional structures of several additional tapasin and TAPBPR molecules. In **Figure 5**, we display the experimentally determined models of human TAPBPR (**Figure 5A**) and human tapasin (**Figure 5G**) as compared with computationally derived models for h-TAPBPR (**Figure 5B**) and h-tapasin (**Figure 5H**) along with examples from other species (TAPBPR - 5C *Pongo abelli* (Sumatran orangutan); 5D *Rattus novegicus*; 5E *Mus musculus*; and 5F *Danio rerio*). Tapasin comparisons are shown as – 5I *Chlorcebus aethiops* (green monkey); 5J *Rattus novegicus*; 5K *Mus musculus*; and 5L *Danio rerio*. The overall structures as expected are remarkably similar, revealing the overall N terminal and IgV domain (from the N terminus to TAPBPR residue 278) and the distinctive C terminal IgC domain (TAPBPR residue 279 to C terminus). Structural distinctions are evident in the amino acid sequence alignment of **Figure 3**. The K22-D35 loop of TAPBPR (as compared to the homologous D12-L18 loop of tapasin) not observed well in the TAPBPR electron density map, is modeled by AlphaFold2 as highlighted by the red dashed oval in **Figures 5A–F**. Residues T106-K111, another region of poor electron density in h-TAPBPR, is modeled as α-helix by AlphaFold2 (**Figures 5B–F**).

**Figure 6:**
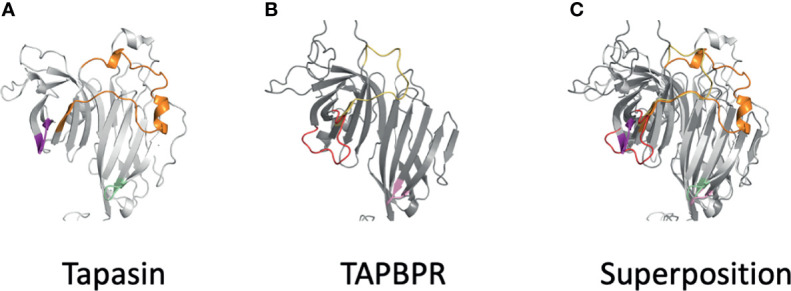
Differences of AlphaFold2 modeled loops of tapasin and TAPBPR suggest distinct details of MHC interactions. **(A)** Loops of tapasin (E12-L18, magenta), (E72-G101), and (Q189-193, pale green) are shown next to **(B)** corresponding loops of TAPBPR (D23-D35, red), (E102-A118, yellow), and (Q209-R213). Superposition the two models is shown in **(C)**.

The Alphafold2 analysis of several tapasin structures reveals modeling for tapasin residues L26-R37 (aligned with L38 to R59 of TAPBPR, **Figure 4**), which was not built into the original tapasin structure (3F8U) because of poor density. AlphaFold2 also recognizes a conserved α-helix (A83 to T91), unique to tapasin, distinct from the corresponding short loop (Q105-T108) of TAPBPR a region poorly defined in TAPBPR. The conserved loop E72-G101 of tapasin is longer than E98-A114 of TAPBPR (see comparison in **Figure 6**).

## Viewing the Future

The importance of understanding the mechanistic details of peptide loading of MHC I molecules, not only with respect to the obvious applied utility of appreciating the cellular evolution of immunevasion in tumorigenesis (60–62), but also with respect to appreciating the interplay between basic aspects of the protein folding problem, peptide loading, and structure prediction cannot be overemphasized. In this brief review we highlight how structural information—derived both experimentally and computationally—complements our understanding of fundamental aspects of immune function. With improved experimental methods [crystallographic, electron microscopic (both cryogenic and tomographic)], expansion of available sequence and structural databases, and remarkable advances in artificial intelligence and computational approaches, we may anticipate not only a host of solutions to vexing, long-standing questions, but may even look forward to deeper and more exciting questions that result from this enlightenment.

## Author Contributions

DM, DT, JJ, LB, JA, MM and KN conceived the concepts and outlined the paper. DM, DT, JJ, LB, and KN designed and prepared figures. All authors wrote and edited the manuscript. All authors contribute to the article and approved the submitted version.

## Funding

This work was supported by the Intramural Research Program of the NIH.

## Conflict of Interest

The reviewer TJ declared a past co-authorship with one of the authors JJ.

The remaining authors declare that the research was conducted in the absence of any commercial or financial relationships that could be construed as a potential conflict of interest.

## Publisher’s Note

All claims expressed in this article are solely those of the authors and do not necessarily represent those of their affiliated organizations, or those of the publisher, the editors and the reviewers. Any product that may be evaluated in this article, or claim that may be made by its manufacturer, is not guaranteed or endorsed by the publisher.
